# Identifying the needs and problems of those left behind, and working with them to address inequities in sexual and reproductive health: a key focus of *Reproductive Health* for 2020

**DOI:** 10.1186/s12978-020-0856-9

**Published:** 2020-01-21

**Authors:** José M. Belizán, Suellen Miller, Venkatraman Chandra-Mouli, Verónica Pingray

**Affiliations:** 1 Department of Mother and Child Health Research, Institute for Clinical Effectiveness and Health Policy (IECS-CONICET), Buenos Aires, Argentina; 2 Safe Motherhood Program, University of California, San Francisco, USA; 3 Department for Reproductive Health Research, World Health Organization, Geneva, Switzerland

One of the key messages of the Sustainable Development Goals is to ensure that no one is left behind in development efforts, and to focus these efforts on those who are most likely to be left behind [1]. We are well aware that there are enormous inequalities and inequities in sexual and reproductive health [2]. The huge differences in the rates of maternal mortality between and within countries, speak to this [3]. What this means is that some individuals, families, groups, communities, and countries are much more likely than others to experience sexual and reproductive health problems, and when they do are less likely to be able to obtain the health and social services they need to overcome these problems and to get back to good health. These inequalities and inequities exist in situations of peace and security, even in high-income countries [4–7]. However, in situations of conflict and natural disasters, they are greatly exacerbated [8–14].

The editorial team of the *Reproductive Health* Journal wants to contribute to efforts to shed light on the sexual and reproductive health needs and problems of the most vulnerable, those most likely to be left behind, and on efforts being made to address inequalities and inequities. Given this, the Journal will prioritize articles that describe efforts addressing such population groups.

We are convinced that solutions must come *from* the most affected populations and *from* those who work with and for them. That is why we will welcome articles that articulate the needs and problem, hopes and expectations, fears and concerns, by members of these populations themselves, and the solutions that they propose. We also welcome articles from individuals who work directly with these populations.

Two other areas will be prioritized in the *Reproductive Health* journal. One is Adolescent Sexual and Reproductive Health and Rights (ASRHR). Adolescents were largely neglected in the context of the Millennium Development Goals [15]. In the context of the Sustainable Development Goals, they are receiving the attention they deserve [16, 17]. Our journal wants to contribute to sharing and learning between adolescents and with researchers, programmers, policy makers, and funders together, and thereby help ensure that focus is put to the best possible use. The second area is the delivery of interventions in the pre-conception period. In an Editorial published in *Reproductive Health* introducing a Supplement on Preconception Care we stated: "The preconception window has been recognized as one of the earliest sensitive windows of human development, and interventions that focus on this period have the potential to affect not only pregnancy but long term outcomes as well" [18]. Given that the preconceptual period has been identified as a critically important stage that influences maternal and perinatal health, interventions that are being developed to improve the coverage of preconceptual care, such as family planning, contraception, nutrition, lifestyle factors (e.g. smoking, alcohol, caffeine, weight) vaccinations, reduction of harmful exposures, prevention and treatment of chronic and infectious diseases, and environmental exposures are of interest [18–20].

We will continue with the two special sections on Female Genital Mutilation/Cutting and on Respectful Care during Childbirth at Health Facilities, as we believe that these issues reflect great inequality and inequity, with powerful implications for reproductive health. Health providers must have an active role to end female genital mutilation/cutting and achieving a respectful care, by adapting or creating behavioral change strategies including their own evaluation of their behavior and its change [21, 22]. As previously stated, these two sections will prioritize the needs, problems, and the solutions developed and implemented to improve them.

In summary, as we move towards the start of the third decade of the twenty-first century, the priority of the *Reproductive Health* journal is to publish contributions highlighting the plight of those who are worst affected by sexual and reproductive health problems, and showcasing actions taken by those affected and those who work with them to overcome this unacceptable situation of inequality and inequity.
